# Regulation of forager honey bee appetite independent of the glucose-insulin signaling pathway

**DOI:** 10.3389/finsc.2024.1335350

**Published:** 2024-02-15

**Authors:** Saleh Ghanem, İrem Akülkü, Kübra Güzle, Zaeema Khan, Christopher Mayack

**Affiliations:** ^1^ Molecular Biology, Genetics, and Bioengineering, Faculty of Engineering and Natural Sciences, Sabancı University, Istanbul, Türkiye; ^2^ US Department of Agriculture, Invasive Species and Pollinator Health Research Unit (ISPHRU), Western Regional Research Center (WRRC) in the Pacific West Area (PWA), Davis, CA, United States

**Keywords:** octopamine, PER assay, biogenic amines, insulin-like-peptide (ILP), energetic homeostasis, *Apis mellifera*

## Abstract

**Introduction:**

To maintain energetic homeostasis the energetic state of the individual needs to communicate with appetite regulatory mechanisms on a regular basis. Although hunger levels indicated by the energetic state and appetite levels, the desire for food intake, tend to be correlated, and on their own are well studied, how the two cross-talk and regulate one another is less known. Insects, in contrast to vertebrates, tend to have trehalose as the primary sugar found in the hemolymph, which could possibly serve as an alternative monitor of the energetic state in comparison to the glucose-insulin signaling pathway, found in vertebrates.

**Methods:**

We investigate how manipulating hemolymph sugar levels alter the biogenic amines in the honey bee brain, appetite levels, and insulin like peptide gene expression, across three age classes, to determine how the energetic state of the honey bee might be connected to appetite regulation.

**Results:**

We found that only in the forager bees, with a lowering of hemolymph trehalose levels, there was an increase in octopamine and a decrease in tyramine levels in the honey bee brain that corresponded with increased appetite levels, while there was no significant changes in *Insulin Like Peptide-1 or 2* gene expression.

**Discussion:**

Our findings suggest that hemolymph trehalose levels aid in regulating appetite levels, in forager bees, via octopamine and tyramine, and this regulation appears to be functioning independent of the glucose insulin signaling pathway. Whether this potentially more direct and rapid appetite regulatory pathway can be generalized to other insects, which also undergo energy demanding activities, remains to be investigated.

## 1 Introduction

An integral part of maintaining energetic homeostasis is the ability for the organism to assess its energetic state and adjust its energy intake accordingly (1). In insects, hunger, the reflection of the need for energy to maintain energetic homeostasis, has been studied extensively, but how this cross-talks with appetite regulation and what physiological factors are involved for the regulation of one another, is less understood (2–4). Flying insects in particular are an ideal model system for understanding appetite regulation and how this cross- talk occurs with the energetic state because their energetic needs vary considerably in a short time frame (5). This is compounded by the fact that insects are typically small in size, restricting their amount energy storage space in terms of fat storage, and on top of this, most have relatively little fat stores for more efficient flying (6). For example, a foraging honey bee is known to obtain just enough nectar to reach its foraging destination and has little to no fat stores, relying on hemolymph trehalose instead, to fuel its flight (7). Thus, to ensure survival, flying insects must be able to rapidly coordinate energy sensing and the behavioral intake of food (5).

Trehalose, as well as glucose and fructose are hemolymph sugars, which are circulating around the body of the honeybee (8, 9), allowing them to provide a platform by which constant monitoring of the energetic can be achieved (10). However, vertebrates do not store energy in the bloodstream in the form of trehalose, which is a nonreducing disaccharide of glucose. In stark contrast, for invertebrates, it is trehalose that is the primary sugar found in the hemolymph and is known to fluctuate the most in order to maintain stable glucose levels (11). For example, holometabolic insects like honeybees (*Apis mellifera*) rely on trehalose, instead of glucose, to provide fuel for their daily life activities (10). As a nonreducing sugar, with low reactivity, it can accumulate in higher concentrations (50-100 mM) in the hemolymph of insects, without toxic effects (12). When the absorption of carbohydrates exceeds what is immediately needed, glycogen and trehalose are produced. When reserves need to be utilized during an energy taxing activity such as insect flight, both are readily converted to glucose for cellular energy (13, 14). In honey bees, trehalose is present in the hemolymph in concentrations from 9.6 to 16.4% of all soluble sugars (15), so it is plausible that honey bees may monitor hemolymph trehalose levels as an indicator of their energetic state and this may be required because it would be more efficient, and direct, than monitoring hemolymph glucose levels via the glucose- insulin signaling pathway, that is involved with the vertebrate appetite regulation system (16).

Biogenic amines in the honey bee brain are likely to serve as the communication bridge between hemolymph trehalose levels and appetite regulation as they have a major impact on the cellular and metabolic characteristics of neurons, altering the effectiveness of pre- to post-synaptic connections. In this way, neuromodulators are the molecular building blocks of all nervous system plasticity. Reciprocal interactions between the nervous system and metabolic or physiological states such as starvation in non-nervous tissues show the long-lasting actions of biogenic amines mediated by different cellular signaling pathways (17). Octopamine and dopamine in particular are known to influence appetite levels in honey bees by altering the sucrose solution food reward perception thresholds. Dopamine causes lower honey bee appetite levels however (18), as this is likely to be more associated with the response to obtaining the food reward from associative learning mechanisms (19). Consequently, there is not much evidence that dopamine is linked to fluctuating hemolymph sugar levels that reflects the energetic state of the individual (17). Although tyramine typically has an opposite effect of octopamine for metabolic regulation (20), tyramine, the pre-cursor to octopamine, was also shown to increase honey bee appetite levels (21). Serotonin on the other hand is more associated with food intake behavior per say and not necessarily appetite regulation based on hemolymph sugar levels. The regulation of food intake is instead coming directly from crop and gut mechano-receptors so when the gut is stretched then there is elevated serotonin in the brain that causes a decrease in food intake and not necessarily appetite levels (22).

Octopamine appears to be the most promising biogenic amine to be involved in appetite regulation that is driven based on the amount of hemolymph sugar levels in insects. This biogenic amine can regulate the mobilization of glycogen and release more trehalose into the hemolymph. This process has usually been noted in the context of the flight or flight response, as its function is analogous to norepinephrine in vertebrates (23, 24). In general, octopamine is associated with metabolic demanding activities such as thermoregulation and the regulation of foraging activity (25–28). Further evidence of the role of octopamine in insect appetite regulation can be found with *Drosophila melanogaster*. An octopaminergic neuronal subcircuit has been identified in the brain of the fruit fly that is directly linked with feeding decisions related to their appetite (29). In fruit flies octopamine also affects hemolymph sugar content, starvation induced hyperactivity, appetite levels and feeding behavior (30–33).

There are large differences in the biogenic amine levels in the honey bee brain as the bee ages (34), so it is likely that their role in appetite regulation may differ across honey bee age classes. In fact, the honey bee forager age class responds the strongest to a starvation treatment in terms of appetite levels and only in this age class was there a significant lowering of glucose, fructose, and trehalose levels in the hemolymph that corresponded with an increase in octopamine levels in the brain (35). This suggests that highly variable hemolymph sugar levels may act as a gauge of the energy state of foraging honeybees. Furthermore, trehalose levels in the hemolymph and octopamine levels in the brain appear to interact with one another that in turn modulates appetite levels in the forager and nurse age class in particular (36). We therefore suspect that the naturally higher levels of octopamine as the bee ages may result in higher sensitivity to fluctuating trehalose levels in the hemolymph as this would result in a more rapid and direct way for the forager bee to assess its energetic state. The need for this as opposed to relying on the glucose-insulin signaling pathway is in line with the fact that foragers tend to exhibit significantly more energetically demanding lives, and their energetic demands change rapidly within a course of a day while foraging for food for the colony (37). Supporting this notion, there are significantly different gene expression profiles of pathways associated with insulin signaling when comparing nurse and forager bees, with a downregulation of insulin signaling pathways in forager bees (38).

Previous work has typically studied these regulatory mechanisms related to the regulation of hemolymph sugar levels on a physiological level, or appetite regulation on a neural level, in isolation. However, it is necessary for the two to cross-talk in order to maintain energetic homeostasis. What remains less understood is how hemolymph sugar levels are possibly controlling appetite regulation in insects and in turn how appetite changes will aid in the regulation of hemolymph sugar levels. For example, we know that octopamine modulates appetite regulation in insects (29), but it is unknown if hemolymph sugar levels can modulate the octopamine levels in the brain and how this is accomplished. Therefore, our aim is to investigate possible neural and physiological mechanisms that are involved in feedback loops between hemolymph sugar levels and appetite regulation, as the bee ages, which are necessary to maintain energetic homeostasis. With the same individuals, across three major age classes, we manipulate the hemolymph sugar levels via thorax sugar injections, measure the insulin like peptide (ILP-1 and ILP-2) gene expression levels, biogenic amine levels in the brain, and appetite levels using the Proboscis Extension Response (PER) assay, to investigate how appetite and energetic state is regulated in the honey bee. Here, we take advantage of using Fucose and Sorbose, which are non-metabolizable sugars and act as a competitive inhibitors of trehalose-p-synthase, that effectively artificially reduce trehalose levels in the hemolymph (36, 39), to better understand its role in appetite regulation. We hypothesize that forager bees specifically, independent of the glucose-insulin signaling pathway, will increase their appetite levels in response to the lowering of hemolymph trehalose levels and this will be due to increased octopamine levels in the brain.

## 2 Materials and methods

### 2.1 Bee preparation and collection

Brood frames from three source colonies of *Apis mellifera anatoliaca* were collected from an apiary on the Sabanci University campus, located in Istanbul, Turkey and placed in an incubator set to 32°C and 60% Relative Humidity. The newly emerged bees were removed within 24 h of hatching, mixed randomly, and marked with a dot of Testors enamel paint (Testors, Vernon Hills, IL, USA), on the back of their thorax. Bees that were harnessed immediately after marking for further experimentation were considered to be in the newly emerged age class, while the rest, around 1,500 individuals, were reintroduced into a hive and re-collected individually using a 20 ml glass vial, 1 week later, that were considered as nurse bees and 4 weeks later that were considered as forager bees.

### 2.2 Appetite assay

Newly emerged, nurse, and forger bees were chilled on ice until immobilized, and harnessed, using a plastic straw and a small piece of duct tape, in groups of 45 individuals. A 30 min waiting period was used to allow the bees to acclimate to the harness. Each group of bees were injected with 1 µl of the following treatments, into the thorax, using a 10 µl Hamilton syringe (Hamilton, Reno, NV, USA), with a 30-gauge needle (Becton, Dickinson and Company, NJ, USA): Ringer’s solution, 1.5 M of trehalose, 3 M glucose, 3 M fucose, 3 M fructose, and 10% sorbose. Appetite was measured using the Proboscis Extension Reflex (PER) assay 10 min after injection. This consisted of touching the antennae of the honey bee with ascending concentrations of sucrose solution (0%, 0.1%, 0.3%, 1%, 3%, 10%, and 30%) and noting whether the bee extended its proboscis in attempt to feed on the sucrose solution, water was used in between each sucrose concentration to desensitize the bee (40).

### 2.3 Hemolymph extraction

Each bee was then unharnessed and pinned on a beeswax plate to extract 1 µl of hemolymph using a 1 µl glass microcapillary (Drummond Scientific Company, PA, USA). Hemolymph was collected after making an incision in between the second and third abdominal segment using a size 0 insect pin (Entosphinx, Pardubice, Czech Republic). The extracted hemolymph was diluted 1:1000 with sterile Millipore (Burlington, MA, USA) filtered water and kept on ice immediately to prevent melanization. A total of 30 µl of this was transferred to another 1.5 mL microcentrifuge tube and this was lyophilized overnight at -40° C.

### 2.4 Validation of hemolymph sugar manipulation

#### 2.4.1 Hemolymph sample derivatization

A total of 10 µl of pyridine with 2.5 mg/ml hexachlorobenzene (internal standard), along with 20 µl of N, O-Bitrifluroacetamide (BSTFA) (Thermoscientific, Waltham, MA, USA) were added to 30 µl of the diluted hemolymph solution (previously lyophilized) inside a 1.5 ml microcentrifuge tube, sealed with parafilm to minimize evaporation. This was then heated to 70° C for 3 hr to allow for the derivatization reaction to go to completion. The mixture was then transferred to a 2 ml amber microvial with a spring loaded 100 µl glass insert.

#### 2.4.2 GC-MS instrument parameters

The hemolymph samples as well as the sugar standards were analyzed using a Shimadzu GC-MS QP-2010 ULTRA (Shimadzu, Kyoto, Japan). The carrier gas used was helium, with a flowrate of 1.3 ml/min. We used a Restek Rtx-5MS column (length 60 m, thickness 0.25 μm, diameter 0.25 μm). The temperature of the injector was set to 280° C, and the mass spectrometer temperature was set to 230° C. Furthermore, the injection temperature was set to 65° C, held for 2 min, increased by 6° C per min, to finally reach 300° C, which was held for 15 min.

Upon running the standards, the peaks were identified by comparing the retention times and the mass spectral ion numbers to the ones reported on PubChem. The peaks of the target sugars (trehalose, fructose, fucose, and glucose) were manually integrated and then they were normalized via the internal standard peak area. There were six different standard mixtures that were run, such that in total there was 6 different concentration levels per sugar. These standards were used to form the standard curves for the quantification of each sugar in mg/ml (41). Based on these validation results we confirmed that we could only successfully independently manipulate trehalose levels using a 10% sorbose injection treatment and glucose using a 3 M glucose injection. Therefore, further experiments only focused on the forager class and the effects of trehalose and glucose level manipulations on the biogenic amine levels in the brain.

### 2.5 Biogenic amine and the insulin-like peptide assays

A total of 90 forager bees for each of the following 1 µl injection treatments: Ringer’s solution, 10% sorbose, and 1.5 M trehalose were collected and harnessed as mentioned above, consisting of 45 bees per trial. The treated honey bees were flash frozen in liquid nitrogen 10 min after injection. Half was separated and stored at -80°C for HPLC analysis for biogenic amine quantification and the other half for insulin-like peptide (ILP-1 and ILP-2) qPCR relative gene expression analysis.

#### 2.5.1 Sample preparation

Bee heads from the biogenic amine assay were dissected on dry ice, the outer chitin layer of the brain was removed. The brain was transferred to a pre-chilled 1.5 ml microcentrifuge tube on dry ice and then stored at -80° C until further analysis. A total of 100 pg/µl of synephrine (internal standard for octopamine and tyramine) and N-methyl serotonin oxalate (internal standard for dopamine and serotonin) were added to 0.2 M perchloric acid. Each brain was added to 50 µl of the internal standard mixture. The samples were then thawed on ice for 50 s and macerated using a sterile pestle. These were then sonicated in ice water for 5 min. After sonification, the samples were chilled on ice for 20 min and then centrifuged at 4°C for 10 min at 15,000 g. The supernatant of each sample was then transferred to a 2 ml dark amber micro vial with a 100 µl glass insert for HPLC analysis.

#### 2.5.2 HPLC analysis

HPLC analysis for quantification of biogenic amines was conducted using a Thermo Scientific Dionex Ultimate 3000 HPLC system consisting of a SR-3000 solvent rack, a refrigerated ISO-3100BM pump, a WPS-3000TBSL analytical autosampler maintained at 5° C, a 150 mm x 2.1 mm high-efficiency C18 reverse phase Acclaim 120 column maintained at 40° C and a 4-channel 6011RS Ultranalytical Cell electrochemical detector (Thermo Fisher Scientific, Waltham, MA, USA). Only two channels were used for detection: one channel was set to 650 mV for octopamine and tyramine, while other was set to 350 mV for dopamine and serotonin. The mobile phase consisted of 7.5 mM sodium dihydrogen monophosphate, 1.7 mM 1-octanesulfonic acid sodium salt, and 25 µM of 100 mM EDTA-tetrasodium tetrahydrate that were added to a 1:10 (v/v) acetonitrile solution. The pH of the mobile phase was adjusted to 3.00 by using phosphoric acid titration. The mobile phase solution was sonicated in ice water for 20 min and then filtered with a 0.2 µm membrane filter paper under vacuum pressure. Mobile phase was kept at 4°C until used. The flow rate of the mobile phase was set to 0.2 ml/min and the injection volume was set to 2 µl. Peaks were integrated using the Chromeleon 7.2.10 software and quantities were calculated on a per brain basis from standard curves. A total of 24 randomized samples were run per day. External standards were included before and after each run (35). All internal and external standards were purchased from Sigma-Aldrich.

### 2.6 Insulin-like-peptide 1 and insulin-like-peptide 2 gene expression assay

The honey bees were macerated using a sterile pestle in 1 ml of one step RNA reagent (Bio Basic, ON, Canada) placed in 1.5 ml microcentrifuge tubes. The RNA was isolated using the EZ-10 spin Column Total RNA Minipreps Super Kit (Bio Basic, ON, CA) by following the manufacturer’s instructions and the amount of RNA was standardized for cDNA synthesis. The cDNA was synthesized according to the manufacturer’s instructions using the OneScript cDNA Synthesis Kit combined with an AccuRT Genomic DNA Removal Kit (Applied Biological Materials, Richmond, Canada). Then, in each well of a 96 well plate (Axygen PCR MICROPLATE), the following was added: 5 μl of master mix (Abm Blastaq Green 2x PCR, Richmond BC, Canada), 1.2 μl of forward and reverse primer of either ILP1 or ILP2 using self-designed primers (*ILP-1*: F – 5’-TGGTCGAACTTTGTCAAGTGCAT-3’, R – 5’-GCAACTCCTCTGTCGTGCAA-3’, *ILP-2*: F– 5’-GGTCGAACTTTGTCAAGTGCAT-3’, R – 5’-TTAACGGGCACCGCAATAGG-3’), 2.8 μl of Wisent multicell nuclease free water, and 1 μl of template cDNA for a final reaction volume of 10 µl. The self-designed primers were first validated using conventional PCR. For the qPCR assay, the following thermocycler program was set on the Roche LightCycler 480 II (Roche, Rotkreuz, Switzerland): quantification for 50 cycles (95° C ramp rate 2.2 for 30 s, 60° C ramp rate 2.2 for 30 s, and 72° C, ramp rate 4.4 for 1 min). Each sample was run in duplicate, if the run was unsuccessful, it was then re-ran again. Relative gene expression was normalized using the RPS5 reference gene (*RPS5*: F – 5’-GATGTTTCTCCGTTACGACGAGT-3’, R – 5’-GAGTTCATCGGCTAAACATTCGG-3’).

### 2.7 Statistical analysis

All analyses were carried out using the R statistical package. Normality of all data was first checked using the Shapiro-Wilk test. The hemolymph sugar levels, biogenic amines, insulin like peptide gene expression were found to be non-normal, so a Kruskal Wallis test was used followed by a pairwise Wilcoxon *post hoc* tests with a Bonferroni correction to account for multiple comparisons. For the hemolymph analysis, the hemolymph sugar level was the dependent variable, while the kind of sugar measured, and the type of sugar injected into the thorax were the independent variables. Comparisons were made within age class and within sugar type measured as our primary interest was validating our ability to manipulate sugar levels using different sugar injections. For the biogenic amine analysis, the amount of biogenic amine served as the dependent variable, while age and sugar injection treatment served as the independent variables. Comparisons were made across age classes and within age classes across the sugar injection treatments. For the insulin-like peptide analysis, normalized gene expression from the -delta delta CT method served as the dependent variable, while gene target and sugar injection thorax treatment served as independent variables. Comparisons were made within and across the thorax injection sugar treatments.

The Gustatory Response Score (GRS) to measure appetite levels was found to be normal, so a Two-Way ANOVA was used followed by a Tukey HSD *post hoc* test for multiple comparisons. The GRS score was the dependent variable, while age and sugar injection treatment served as the independent variables. Comparisons across age were made first and then comparisons of the appetite within age subsequently followed.

## 3 Results

### 3.1 Validation of hemolymph sugar level manipulations within each age class

**Figure 1:**
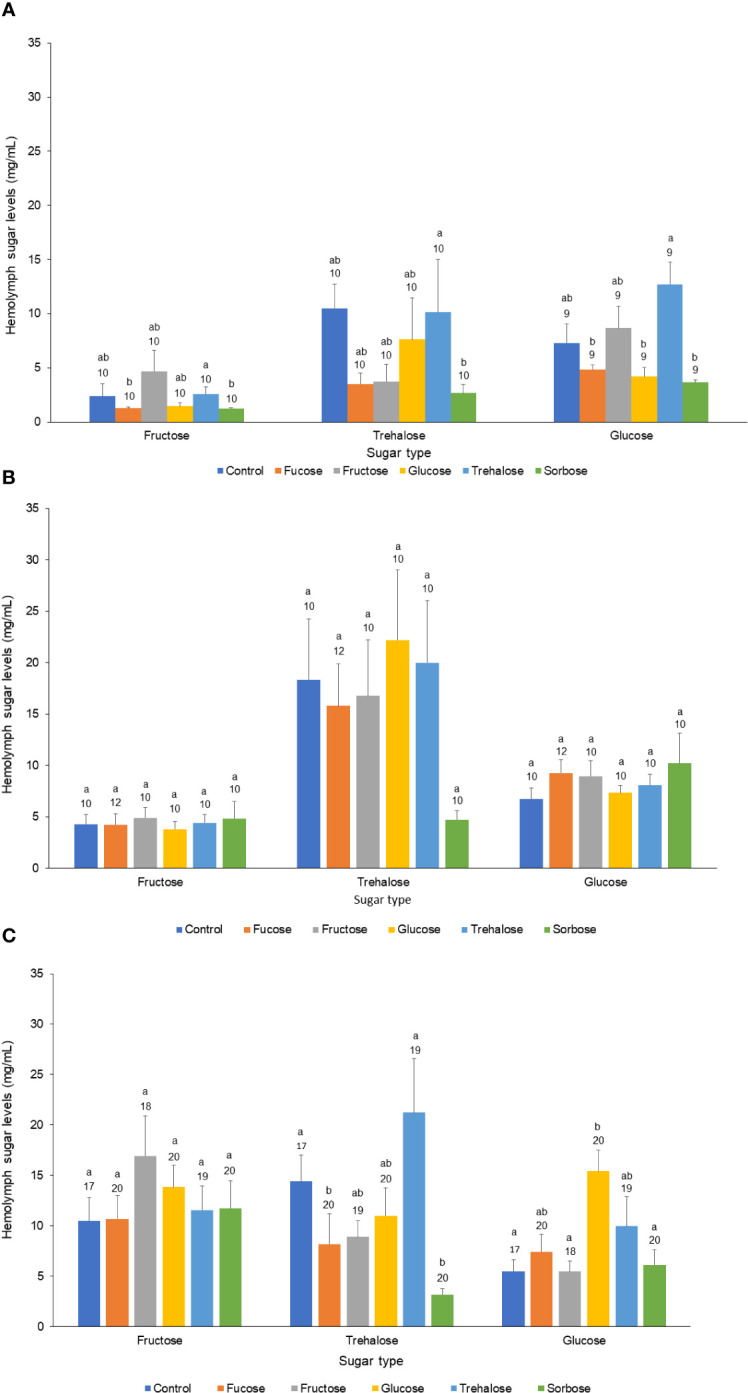
Effect of sugar injection treatment on major hemolymph sugar levels across the three honeybee age classes. The three major hemolymph sugars (Fructose, Trehalose, and Glucose) across all three age classes of honey bees: **(A)** Newly Emerged, **(B)** Nurse, and **(C)** Forager. Each bar represents the mean for each sugar and the bars represent standard errors. The legend and different color of each bar refers to the kind of sugar solution that was injected into the thorax of the bee. The sample sizes are indicated above each bar along with letters denoting significant differences at the 0.05 alpha level.

For the forager age class there was a significant decline in trehalose levels for the fucose and sorbose injected bees, while there was a significant increase in glucose levels of the glucose injected bees (Kruskal Wallis Test: glucose: χ^2^_5,113_ = 14.15, P = 0.015; trehalose: χ^2^_5,113_ = 24.74, P < 0.001). The other sugar levels were not significantly different from the Ringer injected controls. In the nurse and newly emerged age classes, the 1 µl thorax injections did not result in significantly different sugar levels for any of the fructose, glucose, and trehalose hemolymph levels measured in comparison to the Ringer’s control injections (Nurse fructose: χ^2^_5,61_ = 0.95, P = 0.97; Nurse trehalose:: χ^2^_5,61_ = 3.05, P = 0.69; Nurse glucose: χ^2^_5,61_ = 3.38, P = 0.69; Newly Emerged fructose: χ^2^_5,59_ = 11.62, P = 0.04; Newly Emerged trehalose: χ^2^_5,59_ = 11.59, P = 0.04; Newly Emerged glucose: χ^2^_5,59_ = 17.51, P = 0.004) (**Figure 1**).

### 3.2 Appetite levels in relation to sugar injection treatments

**Figure 2:**
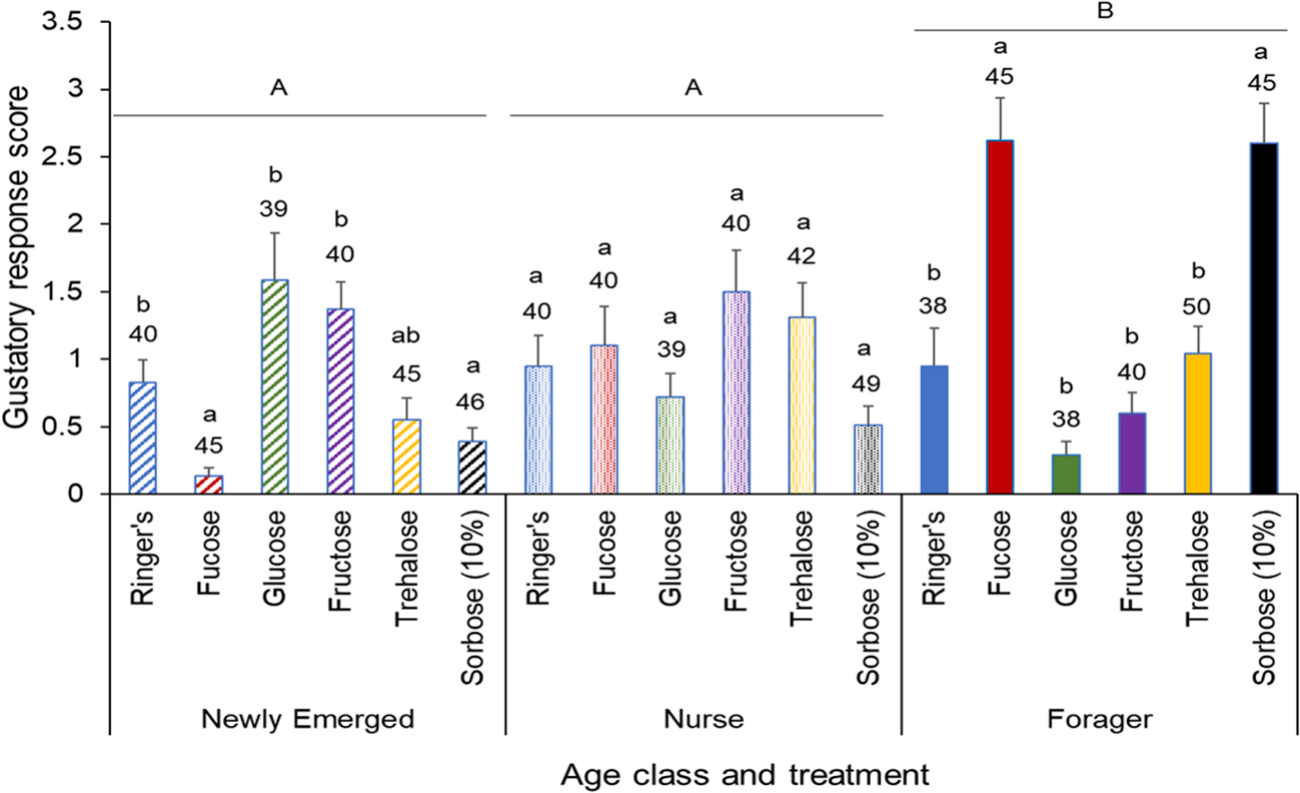
Gustatory response score of different honeybee age classes, with each class subjected to the same 6 treatments. Means and standard errors of the gustatory response scores of each injection treatmentacross three age classes: Newly Emerged, Nurse, and Forager. The six treatments included Ringers solution (treatment), fucose, glucose, fructose, trehalose and sorbose. The sample size of each treatment group is indicated above each bar. The gustatory response score is the sum of the proboscis responses across the PER assay, so a higher the score represents a higher appetite level. The capital letters denote significant differences across the age classes, while the lower case letters denote significant differences within the age classes at the alpha = 0.05 level after adjusting for multiple comparisons.

The effect of the sugar injection treatment on the bees in relation to appetite is dependent upon age (Two-way ANOVA: F_10,745_ = 14.08, P < 0.001). Overall, there were significantly higher appetite levels found with forager bees in comparison to newly emerged and nurse bees. Surprisingly, in comparison to the control, newly emerged bees injected with fucose and sorbose had significantly lower appetite levels, while the foragers had a significantly higher appetite levels. There was no significant difference in appetite among the nurse bees from any sugar injection treatment (F_2,745_ = 11.75, P < 0.001; **Figure 2**).

### 3.3 Biogenic amine profiles in relation to forager bee sugar level manipulations

**Figure 3:**
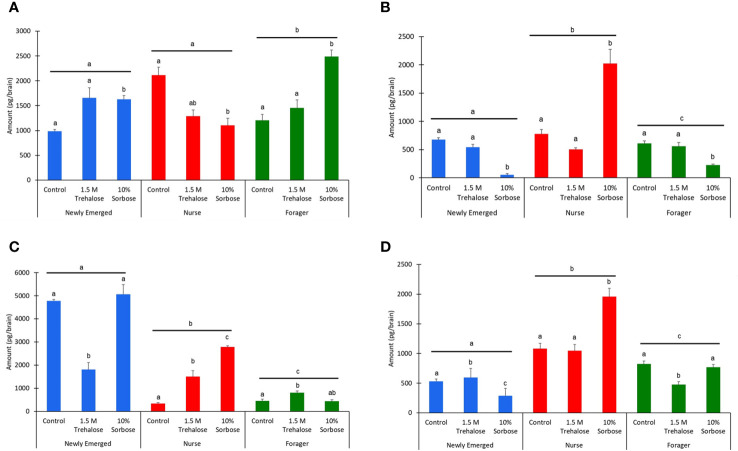
The biogenic amine levels on a per brain basis of **(A)** Octopamine, **(B)** Tyramine, **(C)** Dopamine, and **(D)** Serotonin, across the three honey bee age classes: newly emerged (blue), nurse (red), and forager bees (green). The bars represent means while the error bars represent standard errors. The sample sizes are indicated above each bar. The letters above each bar represent significant differences across the treatments, while the different letters above the lines represent significant differences across the age classes at the alpha = 0.05 level.

The biogenic amine levels are age dependent (Kruskal Wallis test: Octopamine: χ^2^_2,398_ = 15.18, P < 0.001; Tyramine: χ^2^_2,398_ = 24.47, P < 0.001; Dopamine: χ^2^_2,398_ = 95.23, P < 0.001; Serotonin: χ^2^_2,398_ = 129.27, P < 0.001). The 10% sorbose sugar injections caused a significant increase in octopamine levels in the newly emerged and forager bees, but a significant decrease in the nurse bees (χ^2^_2,398_ = 27.78, P < 0.001; χ^2^_2,398_ = 33.70, P < 0.001; χ^2^_2,398_ = 21.73, P < 0.001; **Figure 3A**). The opposite trend was found for tyramine where there was a decrease in newly emerged and forager bees injected with 10% sorbose, but an increase in the nurse bees (χ^2^_2,398_ = 65.84, P < 0.001; Tyramine: χ^2^_2,398_ = 28.98, P < 0.001; Tyramine: χ^2^_2,398_ = 8.12, P = 0.02; **Figure 3B**). There was a significant decrease in brain dopamine levels in newly emerged bees injected with 1.5 M trehalose (χ^2^_2,398_ = 41.75, P < 0.001), but an increase forager bees (χ^2^_2,398_ = 19.18, P < 0.001; **Figure 3C**). Interestingly, only in the nurse bees was there an increase in dopamine from the 1.5 M trehalose injection, but an even higher increase in dopamine levels from the 10% sorbose injection (χ^2^_2,398_ = 61.72, P < 0.001; **Figure 3C**). In newly emerged bees there was a significant increase in serotonin levels for 1.5 M trehalose injected bees, while there was a decrease in the 10% sorbose injected bees (χ^2^_2,398_ = 47.04, P < 0.001). For nurse bees, there was a significant increase only with the 10% sorbose injected bees (χ^2^_2,398_ = 20.64, P < 0.001). In forager bees there was a significant decrease in the 1.5 M trehalose injected bees (χ^2^_2,398_ = 29.21, P < 0.001; **Figure 3D**).

### 3.4 Insulin-like peptide gene expression levels

**Figure 4:**
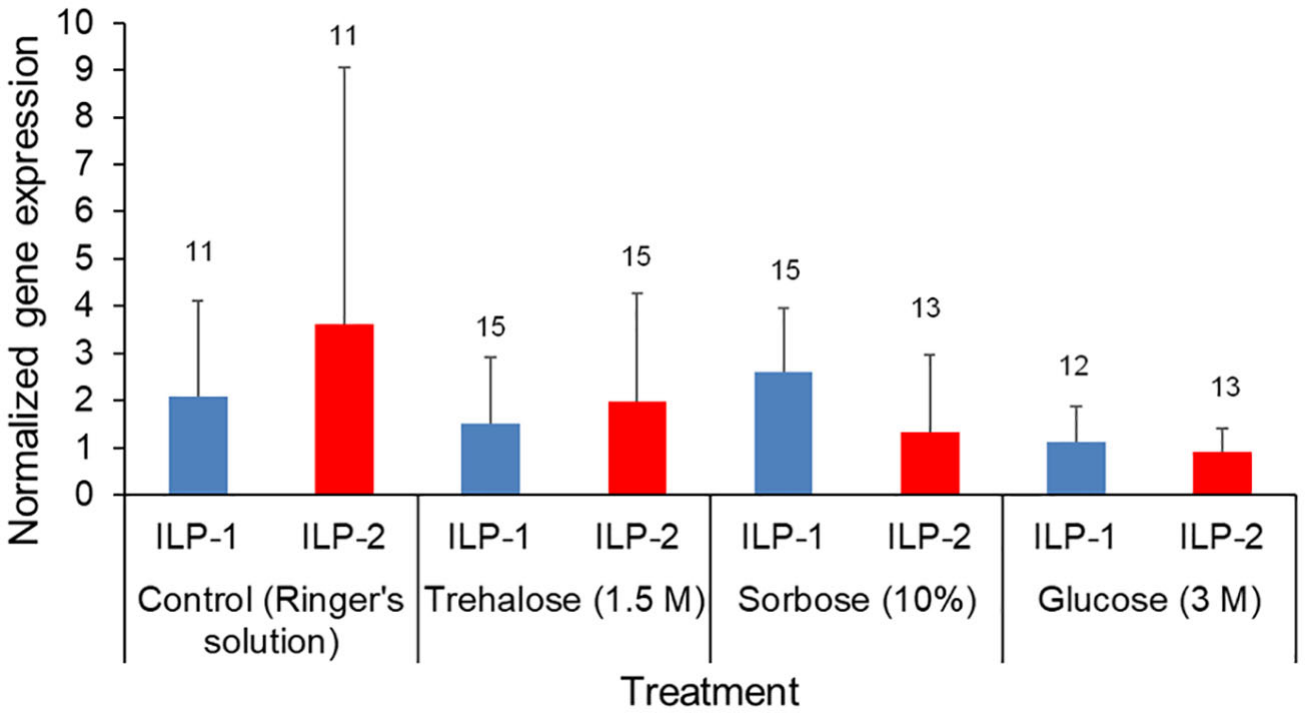
Forager bee qPCR gene expression levels of ILP-1 and ILP-2. The foragers were injected with Ringer’s solution (control), Trehalose (1.5M), Sorbose (10%) and Glucose (3M). Insulin-like protein (ILP)-1 and ILP-2 gene expression was quantified via qPCR, Means and standard deviations of ∆∆CT values representing the normalized relative gene expression of ILP-1 (blue) and ILP-2 (red). The sample sizes are indicated above each bar.

The qPCR gene expression analysis of ILP-1 and ILP-2 indicates there were no significant differences in response to a 1.5 M trehalose, 3 M glucose, or a 10% sorbose injection (Kruskal Wallis test by treatment: χ^2^_3,103_ = 1.18, P = 0.76). There was also no significant differences of gene expression between *ILP-1* and *ILP-2* across the treatments (Kruskal Wallis test by gene target: χ^2^_1,103_ = 0.75, P = 0.39; **Figure 4**).

## 4 Discussion

From the sugar injections we only successfully manipulated glucose and trehalose levels in the forager age class in comparison to the control treatment. This suggests that the newly emerged and nurse bees are likely to have more extensive hemolymph sugar buffering and regulatory capacity in comparison to the forager age class that is independent from changing appetite levels. This perhaps explains why in previous studies the newly emerged and nurse age class appetite levels did not change significantly in response to thorax sugar injections or starvation treatments (35, 36). Corresponding with successful hemolymph sugar manipulations, we found that only forager bees have significantly higher appetite levels when treated with sorbose and fucose, which corresponds with a significant lowering of hemolymph trehalose levels. Interestingly, significantly higher hemolymph glucose levels did not result in a depression of appetite levels in the forager bees.

The nurse bees did not have any significant differences in appetite levels and this could be due to the fact that we could not cause any significant changes in their hemolymph sugar levels from our sugar injections into the thorax. Surprisingly, in the newly emerged bees the fucose and sorbose treatments had the opposite effect compared to forager bees, there was significantly lower appetite levels in these treated bees in comparison to the control treatment. Based on our observations these treatments may have caused a toxic effect in these bees and actually reduced their ability to respond to any stimuli (including water) presented to them during the PER appetite assay. Across age classes however, our findings agree with previous findings, where forager bees had higher appetite levels in comparison to nurse and newly emerged bees (35, 36). Forager bees have a lower sucrose sensitivity threshold and higher metabolic rates in comparison to nurse and newly emerged bees, which is likely to explain their higher overall appetite levels (42). It is also interesting to note that the higher appetite levels in the forager age class are primarily driven by the injection treatments that are responsible for lowering hemolymph trehalose levels, fucose and sorbose.

Among the foragers, injections of sorbose, that resulted in a significant lowering of hemolymph trehalose levels, were linked to notably elevated octopamine levels and reduced tyramine levels, while dopamine and serotonin were not significantly changed. We therefore suspect, as previously suggested (10, 43), that trehalose is the key sugar in the hemolymph which is used to monitor the energetic state of forager bees and there is a feedback loop with octopamine and tyramine levels in the honey bee brain. For example, when hemolymph trehalose levels are low there is an increase in feeding behavior to elevate the hemolymph trehalose levels once again from the synthesis of two glucose molecules by trehalose-p-synthase coming from the ingested food (7). Our findings also suggest that it is very likely that the increase in octopamine and the lowering of tyramine play a role in the increased appetite of the forager bee. It’s worthwhile noting that higher trehalose levels in forager bees did not result in lowering of octopamine levels, suggesting that there may be a signaling pathway for the lowering of the trehalose to increase appetite, but not a mechanism having the opposite effect, when trehalose levels are high. This finding agrees with a previous study that showed in forager bees that both a lowering in hemolymph trehalose levels, plus an increase in octopamine levels, was required to cause a significant increase in appetite levels. Fumurate on the other hand, an octopamine inhibitor, did not result in decreased appetite in bees with lowered hemolymph trehalose levels (36). In addition, when foragers were injected with trehalose, there was no significant depression in appetite. Corresponding to this, there was a significant increase in dopamine and lowering of serotonin levels in the honey bee brain. This suggests that if there is a potential lowering of appetite levels in forager bees it is likely to be modulated by these biogenic amines. Higher serotonin levels for example corresponds with higher feeding levels (22) and higher dopamine levels can result from obtaining a food reward (19). Octopamine can increase hemolymph glucose levels in honey bees, but whether the increased glucose levels comes from the breakdown of trehalose or glycogen, remains unknown. If the honey bee was starved or fully satiated, then octopamine injections did not have an effect on appetite levels (17).

For the newly emerged bees, even though there was a lowering of appetite levels due to the sorbose treatment, the octopamine and tyramine biogenic amine profile of these treated bees matched the forager bees that showed an increase in appetite levels. However, there was an opposite trend for dopamine and serotonin levels in the bees treated with elevated and lowered hemolymph trehalose levels. These findings suggest that the neuronal mechanisms involved in appetite regulation are likely to be age class specific in honey bees. As expected, we did observe overall significant differences in the biogenic amine levels across the age classes. In nurse bees, there are other changes in biogenic amine levels in association with the thorax sugar injection treatments, but none of these can be linked to significant changes in appetite levels. Very little appetite fluctuations seem to be occurring in general in the nurse and newly emerged bee class that are based on hemolymph sugar levels and this is in agreement with previous studies (35). This, may be due to the fact that bees in this age class simply do not have sensitive enough sucrose perception that is expected within the PER paradigm that is precise enough to detect changes in appetite in these age classes (44). Supporting this notion, is the fact that there are significantly higher levels of all biogenic amines in forager bees except for tyramine. Therefore, the forager bees appetite levels may be more sensitive to changes in hemolymph trehalose levels because they tend to have higher baseline octopamine levels and lower tyramine levels in the brain in comparison to nurse and newly emerged bees, which corresponds to the higher sucrose sensitivity in forager bees (34). If this is the case, then it is plausible that forager bees have a more precise mechanism for regulating appetite and are more in tune with their energetic needs, and this would make adaptive sense as they have less energy storage and a higher metabolic rate, resulting in less starvation buffering capacity in comparison to nurse and newly emerged bees (45).

There were no significant differences in gene expression for both ILP-1 and ILP-2 across the 10% sorbose, trehalose (1.5M), and glucose (3M) hemolymph injections for forager bees. We therefore suspect that a lowering of trehalose levels from a sorbose injection or the elevation of trehalose or glucose levels in the hemolymph from injections are not linked to the production of ILP-1 and ILP-2 in forager honey bees and these insulin-like peptides are serving other regulatory roles in honey bees besides appetite regulation. The functional role of these peptides in relation to metabolism in honey bees are still being elucidated (46). Our results suggest that the increase in appetite observed due to reduction of trehalose, resulting from the 10% sorbose treatment, that corresponds with an increase in octopamine and a decrease in tyramine in the honey bee brain, are independent from the glucose-insulin signaling pathway. The production of insulin, or for insects, known as insulin-like peptides (ILPs), are increased by food consumption or additional nutrient storage, while the synthesis of glucagon or the insect counterpart, adipokinetic hormone (AKH), is suppressed. However, it is suspected that honey bees have lost functionality of adipokinetic hormone (AKH) in this context due to relaxed selection from having abundant food resources available on a regular basis inside the bee hive (47). Indeed, under starvation conditions there is no response in AKH gene expression in forager honey bees (48) and very little to no amounts were detected across all three age classes (35). Therefore, there is no functional counterpart to ILP-1 and ILP-2 for the regulation of hemolymph sugar levels in forager honey bees. *AmIlp1* was affected by different diet feeding compositions, but not *AmIlp2* or the *insulin receptor substrate* (*IRS*), further supporting that the latter two are not likely to be involved in metabolic functions (49).

Previously from a *vitellogenin* (*Vg*) double knockdown there was an increase in Juvenile Hormone (JH), appetite, hemolymph glucose and trehalose levels, with a lowering of *ILP-1*, but not *ILP-2* expression, suggesting that there may be a connection between sugar levels, appetite, and *ILP-1* (50). However, Vg is known to be a master regulator that affects many different pathways related to aging, reproduction, and division of labor, so alternative pathways may be responsible for the correlations observed. Forager bees do generally have higher expression of *ILP-1* and *IRS* in comparison to nurse bees and when colonies are chronically starved there is accelerated behavioral maturation that is associated with higher levels of *ILP-1* (38), but based on our results the connection between starvation and *ILP-1* is more likely due to the chronic nature of the starvation where lipid metabolism is likely to be involved. *AmIlp1* expression is found in the fat body of the honey bee and is thought to be more directly related to lipid metabolism. Honey bee lipid stores are also associated with the division of labor in honey bees (51, 52) and here we do not find any large differences in *ILP* expression in a short time frame where we are only manipulating hemolymph sugar levels, so we suspect that ILP-1 is not involved in appetite regulation due to acute starvation. Further supporting this notion, ILP functioning is also known to be associated with behavioral maturation in the honey bee (46). However, our findings do not exclude the possibility of insulin influencing honey bee appetite as it has been shown to increase the appetite of younger and winter bees, but decreases appetite in forager bees (53, 54). A connection with hemolymph sugar levels does not appear to be the case however, as a knockdown of both *ILPs* did not result in influencing hemolymph sugar levels (55).

Adult forager honey bees consume primarily carbohydrates and use them as their major source of energy, and hemolymph trehalose is known to be the main source of fuel to sustain flight when foraging because they have less glycogen and lipid reserves to rely upon (7). To prevent starvation and increase flight efficiency, it is vital for foraging bees to tightly and rapidly regulate both their feeding behavior and sugar metabolism (5). We show that increased octopamine and decreased tyramine levels respond to a lowering of hemolymph trehalose levels, specifically in the forager age class, that corresponds to an increase in appetite levels, that appears to be independent of the glucose- insulin signaling pathway. The feedback loop we established is likely to be more direct and therefore more rapid instead of involving the glucose-insulin signaling pathway, which is more responsive to differences in diet nutrients and chronic starvation. This newly recognized feedback loop is only found in forager bees, which is the age class that would require a more rapid response to changes in energy demands. Additional investigation is needed however, to further elucidate the direct or indirect connections between appetite, insulin, and hemolymph sugar levels.

## Data availability statement

The datasets presented in this study can be found in online repositories. The names of the repository/repositories and accession number(s) can be found below: https://datadryad.org/stash, DOI 10.17605/OSF.IO/ANBSP.

## Ethics statement

Ethical approval was not required for the study involving animals in accordance with the local legislation and institutional requirements because the honey bee samples used in this study were owned by the senior author and were placed on university property.

## Author contributions

SG: Data curation, Formal analysis, Investigation, Methodology, Validation, Visualization, Writing – original draft, Writing – review & editing. İA: Data curation, Formal analysis, Investigation, Methodology, Visualization, Writing – review & editing. KG: Data curation, Formal Analysis, Investigation, Methodology, Visualization, Writing – review & editing. ZK: Funding acquisition, Investigation, Supervision, Writing – original draft, Writing – review & editing. CM: Conceptualization, Data curation, Formal analysis, Funding acquisition, Investigation, Methodology, Project administration, Resources, Software, Supervision, Validation, Visualization, Writing – original draft, Writing – review & editing.
